# 

**Published:** 1907-12-15

**Authors:** 


					﻿Dental
Register
NELVILLE S. HOFF, Editor,
Ann Arbor, Mich.
Vol. LXI— DECEMBER, 1907— No. 12
SAMUEL A. CROCKER & CO., Publishers
OHIO DENTAL AND SURGICAL DEPOT
35 W. FIFTH STREET, CINCINNATI, 0.
PRICE, ONE DOLLAR A YEAR
Entered at the Posloffice at Cincinnati, O., as second-class mail matter
				

